# Characterization of soils conducive and non-conducive to Prunus replant disease

**DOI:** 10.1371/journal.pone.0260394

**Published:** 2021-12-10

**Authors:** Abdur R. Khan, Wisnu A. Wicaksono, Natalia J. Ott, Amisha T. Poret-Peterson, Greg T. Browne

**Affiliations:** 1 Department of Plant Pathology, University of California, Davis, California, United States of America; 2 USDA-ARS Crops Pathology and Genetics Research Unit, Davis, California, United States of America; COMSATS University Islamabad - Abbottabad Campus, PAKISTAN

## Abstract

Successive orchard plantings of almond and other *Prunus* species exhibit reduced growth and yield in many California soils. This phenomenon, known as Prunus replant disease (PRD), can be prevented by preplant soil fumigation or anaerobic soil disinfestation, but its etiology is poorly understood and its incidence and severity are hard to predict. We report here on relationships among physicochemical variables, microbial community structure, and PRD induction in 25 diverse replant soils from California. In a greenhouse bioassay, soil was considered to be “PRD-inducing” when growth of peach seedlings in it was significantly increased by preplant fumigation and pasteurization, compared to an untreated control. PRD was induced in 18 of the 25 soils, and PRD severity correlated positively with soil exchangeable-K, pH, %clay, total %N, and electrical conductivity. The structure of bacterial, fungal, and oomycete communities differed significantly between the PRD-inducing and non-inducing soils, based on PERMANOVA of Bray Curtis dissimilarities. Bacterial class MB-A2-108 of phylum Actinobacteria had high relative abundances among PRD-inducing soils, while Bacteroidia were relatively abundant among non-inducing soils. Among fungi, many ASVs classified only to kingdom level were relatively abundant among PRD-inducing soils whereas ASVs of *Trichoderma* were relatively abundant among non-inducing soils. Random forest classification effectively discriminated between PRD-inducing and non-inducing soils, revealing many bacterial ASVs with high explanatory values. Random forest regression effectively accounted for PRD severity, with soil exchangeable-K and pH having high predictive value. Our work revealed several biotic and abiotic variables worthy of further examination in PRD etiology.

## Introduction

In order to maintain economical yields, *Prunus* orchards (i.e. almonds and other stone fruits) require removal and replanting every 10–20 years. After planting, 3 years of orchard growth are required to bear harvestable crops, and another 3 or more years are needed to reach peak annual yields. Eventually, following 5 to 15 years of peak production, yields gradually decline, ultimately requiring orchard replanting to restore productivity. However, successive orchard plantings of *Prunus* species are subject to complex replant problems that cause serious growth and yield suppression in certain soils. The renewed vigor provided by replanting an orchard can be compromised by these replant problems, some of which are understood and others poorly characterized. For example, several species of phytopathogenic nematodes (PPN) reproduce on *Prunus*, damaging roots, reducing tree vitality, and inducing long-term decline [1]. Also, even in the absence of phytopathogenic nematodes, soils with a recent history of *Prunus* sp. cultivation often mediate what is referred to as “Prunus replant suppression” or “Prunus replant disease” (PRD). The disease, though incompletely understood, is manifested by stunted tree growth and delayed crop productivity [2, 3].

There is a need for a clearer understandings of replant problems and approaches for managing them with less dependence on soil fumigation. Although soil fumigation has been used widely and effectively to manage both PPN and PRD, the practice is increasingly regulated and is not always an option in California orchards. Anaerobic soil disinfestation (ASD), a process based on microbial utilization of organic carbon and nutrient sources, is technically effective for management of PRD [4] but will require further optimization before it is widely feasible. In the meanwhile, integrated pest management strategies—typically based on detailed knowledge of economic crop loss thresholds, disease biology, and environmental interactions, have been used to reduce pesticide usage for agricultural pests and could be improved for managing PPN and PRD with less fumigant. There are general guidelines for assessing the need for preplant soil fumigation based on counts of PPN populations in soil and root samples and on soil texture [5]. However, PRD risk assessments are complicated by the lack of literature convergence on its causes, and there is no clear understanding of how soil environmental variables may influence PRD development or severity.

Literature concerned with PRD biology has been, until recently, based mostly on culture-based inquiries, sometimes coupled with orchard trials and sampling. These approaches have revealed the crop-specific nature of PRD; for example, it affects *Prunus* spp. planted after *Prunus* sp., but not *Prunus* spp. planted after apple [6], citrus, or grape (Browne, *unpublished*). Also, inconsistent evidence has been presented for diverse modes of PRD mediation, ranging from suppressive levels of cyanogenesis from peach root residues [7] to direct pathogenesis by species of fungi and oomycetes [8, 9]. Regardless, the traditional culture-based approaches have offered limited views into soil microbial communities and have been complicated by logistical challenges [10]. Next generation sequencing (NGS) of marker genes has afforded comparatively deeper and more adaptable approaches to characterizing the microbial communities [11, 12]. There are recent examples of NGS applications with high relevance to PRD, including examinations of apple replant disease [13, 14], rose replant disease [15], and peach replant suppression [3, 16]. However, none of the previous studies using NGS examined PRD incidence or severity among multiple soils or related them to soil physicochemical properties.

Here, we report on the use of a soil bioassay to examine relationships of physicochemical and microbial variables in soil to induction of PRD. Among 25 replant soils, we wanted to determine (1) how key soil physicochemical variables related to composition of the soils’ microbial communities and (2) how combinations of certain microbial and/or soil physicochemical parameters related to the soils’ capacities to induce PRD. For these purposes, we selected replant soils with different perennial crop histories and supposed potentials to induce PRD, including: (1) soils that had recently hosted a *Prunus* sp., (2) soils that had hosted a *Prunus* sp. but were subsequently fumigated or subjected to anaerobic soil disinfestation (ASD), and (3) soils from grape vineyards. Data from multiple almond orchard replant trials with fumigated and non-fumigated plots (Browne, *unpublished*) had indicated that replant soils vary in PRD induction capacity, and additional trials demonstrated that both ASD and fumigation prevented PRD induction [4, 17]. Grape vineyard soils were of interest because they are not known to induce PRD and many of them are being transitioned to almond production in California.

## Materials and methods

### Soil sampling

Twenty-five sets of soil samples were collected in spring 2015 from 20 different managed blocks of land distributed across northern, central, and southern portions of California’s Central Valley (Table 1). GPS locations of the sampling locations are provided (S1 File). The managed blocks were all either on private land or University of California (UC) research property, and permission for sampling was obtained for all blocks. Each block was a contiguous field area. Four of the blocks were subdivided into zones according to previous soil treatments or soil series classifications. Collectively, the blocks and zones represented: soils that had recently hosted an almond or other stone fruit orchard (n = 20), soils treated with fumigation or ASD after clearing of a *Prunus* sp. (n = 3), and soils from vineyards (n = 2). In each block or zone, soil samples were collected at three or four random sampling locations. At each sampling location, an 8-cm-diameter hand auger was used to collect soil at two to three points within several meters from depths of 10 to 61 cm. The soil from each sampling location was pooled and mixed. Single 100-mL portions of the mixed soil at each sampling location were frozen on dry ice immediately for subsequent long-term storage at -80 °C, DNA extraction, and microbial community analyses. Additional portions of the sampled soil were used for analysis of soil physicochemical variables, nematode assays, and PRD induction bioassays as described below; these portions were pooled across sampling locations that were within managed blocks or zones before analysis.

**Table 1:** Background information and selected physicochemical properties of California soils examined for relationships to Prunus replant disease.

Soil no.	County-site^a^	Crop history^b^	Field soil trt.^c^	Soil texture	Sand (%)	Silt (%)	Clay (%)	pH	EC (dS/m)^d^	Mg (meq/L)^d^	N total (%)^d^	Exch-K. (meq /100 g)^d^
1	Butte-1	Ald	None	Clay loam	36	37	27	7.81	0.77	3.19	0.058	0.46
2	Butte-2	Ald	None	Sandy loam	55	30	15	7.91	0.82	2.68	0.056	0.71
3	Butte-3	Ald	None	Sandy loam	63	28	9	7.95	0.96	3.02	0.054	0.3
4	Butte-4A	Ald	None	Loam	41	37	22	7.08	0.7	2.7	0.094	0.39
5	Butte-4B	Ald	None	Clay loam	41	32	27	6.95	0.55	1.99	0.09	0.34
6	Colusa-1	Ald	None	Sandy loam	65	22	13	5.75	0.81	1.81	0.037	0.19
7	Colusa-2	Ald	None	Sandy loam	61	27	12	5.61	1.44	4.65	0.041	0.24
8	Merced-1A	Ald	None	Sand	91	9	1	6.34	1.07	2.38	0.02	0.08
9	Merced-1B	Ald	Fum	Sand	92	8	1	6.8	0.5	1	0.02	0.06
10	Fresno-1	Ald	None	Sandy loam	77	16	7	7.85	2.98	2.39	0.038	0.65
11	Fresno-2	Gra	None	Sandy loam	62	32	6	7.34	0.59	1.41	0.024	0.13
12	Fresno-3	Gra	None	Sandy loam	57	35	8	7.57	0.6	1.21	0.029	0.16
13	Fresno-4A	Pe	None	Sandy loam	66	28	6	7.55	1.81	3.73	0.024	0.13
14	Fresno-4B	Pe	Fum	Sandy loam	66	29	5	7.12	1.69	3.93	0.026	0.13
15	Fresno-4C	Pe	ASD	Sandy loam	68	26	6	6.43	1.26	3.46	0.03	0.16
16	Fresno-5	Ne	None	Sandy loam	78	15	7	6.8	1.04	2.76	0.021	0.2
17	Fresno-6	Pe	None	Sandy loam	73	19	8	7.28	2.94	10.17	0.033	0.17
18	Fresno-7	Plu	None	Sandy loam	55	42	3	6.79	1.62	7.13	0.027	0.15
19	Fresno-8A	Ald	None	Sandy loam	71	24	5	6.18	1.02	3.12	0.028	0.13
20	Fresno-8B	Ald	None	Sandy loam	70	22	8	6.68	0.78	3.09	0.026	0.24
21	Tulare-1	Ne	None	Sandy loam	68	23	9	7.6	1.29	1.92	0.032	0.2
22	Kern-1	Ald	None	Sandy loam	86	10	4	6.07	1.78	1.53	0.02	0.11
23	Kern-2	Ald	None	Sandy loam	72	17	11	7.57	1.99	1.08	0.02	0.3
24	Kern-3	Ald	None	Sandy loam	66	18	16	7.68	3.3	5.98	0.039	0.25
25	Kern-4	Ald	None	Sandy loam	45	31	24	7.79	3.02	4.34	0.062	0.34

^a^ Name of county is followed by a number indicating the managed block of contiguous land, and in some cases by a letter code, indicating a zone with a different soil texture or soil treatment.

^b^ Abbreviations indicates crop to which soil was devoted, “Ald” for almond trees, “Gra” for grape vineyard, “Pe” for peach trees, “Plu” for plum trees, and “Ne” for nectarine trees.

^c^ Abbreviations: “None”, “Fum”, and “ASD” represent soils that received field treatments of control (non-treated), fumigation, or anaerobic soil disinfection, respectively.

^d^ Abbreviations: EC = electrical conductivity, Mg = soluble magnesium in saturated paste extract, N = total nitrogen, Exch-K = exchangeable potassium.

### Assessment of PRD induction potential

Each of the 25 soils was assessed for its capacity to induce PRD in a greenhouse bioassay. The soils were separately mixed with autoclaved sand (2:1, soil:sand, v:v) to facilitate adequate soil water drainage. The mixtures were apportioned to three preplant treatments: a non-treated control, fumigation with chloropicrin, and pasteurization with steam. The control portions were stored at 15 to 22°C in shaded, vented polyethylene bags. The portions to be fumigated were placed in polyethylene bags in 19-liter buckets (one bag and bucket per soil) that had been lined with a totally impermeable film (TIF) (Raven, Sioux Falls, SD, USA). The soil in each bucket was injected with 3 ml of chloropicrin (TriCal, Inc.; Hollister, CA, USA), quickly sealed in the bag and TIF, and then, 1 week after treatment, allowed to vent thoroughly. Soil to be pasteurized was injected with steam through multiple nozzles at the bottom of 18-liter metal containers to maintain soil temperature at ≥80°C for 30 min, then allowed to cool.

The treated and control portions of each soil were distributed to 0.9-L pots in a randomized complete block design. There were six blocks, each containing one pair of pots (i.e., paired pots were subsamples) per treatment-soil combination. Each pot was planted with a single recently sprouted peach seedling (*Prunus persica* × *P*. *davidiana* ‘Nemaguard’, a common rootstock for stone fruits and nuts; Sierra Gold Nursery, Inc., Yuba City, CA, USA). The plants were grown in a greenhouse with air temperatures of 16 to 30°C and potted soil temperatures of 17 to 29°C. All plants were watered daily as needed with a modified Hoagland’s solution [18].

Ten weeks after planting, the seedlings were assessed for growth. Plants were washed free from soil, and top and root fresh weights were measured. The plant data were subjected to analysis of variance (ANOVA) using PROC MIXED of SAS Version 9.4 (SAS, Cary NC, USA). The model statement for PROC MIXED specified plant top, root, and total fresh weights as a function of preplant soil treatment, soil number, and interaction of preplant soil treatment × soil number; block (i.e., in the greenhouse experiment) was specified as a random variable. Plant fresh weight means were separated according to 95% confidence intervals. Soils were considered to be conducive to PRD (henceforth “PRD-inducing”) if preplant soil fumigation and pasteurization significantly increased plant top fresh weights, compared to the control. Soils were considered to be non-conducive to PRD (henceforth “non-inducing”) if neither preplant treatment significantly improved growth. The ratio of plant fresh weight (total or top) from control soil divided by the average plant fresh weight in fumigated and pasteurized portions of the soil was calculated and is henceforth referred to as the “control proportion” of plant growth; the smaller the control proportion, the greater the intensity of PRD. The control proportions were determined for plant top and total fresh weights.

### Assessment of physicochemical and nematode variables

Soil physicochemical analyses were performed by the UC Davis Analytical Lab using described methods (https://anlab.ucdavis.edu/methods-of-analysis). Assays of the soil for selected phytopathogenic and free-living nematodes were performed by Nematodes Inc., Selma, CA; nematodes were extracted using the centrifugal-sugar flotation technique [19]. The extracted nematodes were identified according to morphology and counted.

The soil physicochemical variables determined were clay (%), sand (%), silt (%), pH, electrical conductivity (EC, dS/ms), soluble calcium (Ca, meq/L), soluble sodium (Na, meq/L), soluble magnesium (Mg, meq/L), total N (%), and exchangeable potassium (K) (meq/100 g soil) (Table 1, S1 File). Concentration of soluble Ca, Na, and Mg were used to calculate the sodium adsorption ratio (SAR) and the exchangeable sodium percentage (ESP) of soils. The physicochemical variables were checked for covariance, and only one variable was chosen from pairs of variables showing correlation of *ρ* ≥ 0.85. The variables clay (%), sand (%), pH, electrical conductivity (EC), total N (%), soluble magnesium (Mg) (meq/L), and exchangeable potassium (K) (meq/100 g soil) were retained. These variables were square root transformed, then normalized by subtracting the mean and dividing by the standard deviation across samples for each variable. Principal Component Analysis (PCA, in PRIMER version 7, henceforth PRIMER7 [20]) of the normalized physicochemical variables was used to ordinate the 25 soils. Pearson correlations were determined between the selected soil physicochemical variables and the control proportions for plant top weights in the bioassay. To examine associations between nematode populations and PRD induction potential, permutational multivariate analysis of variance (PERMANOVA, in PRIMER7) was conducted on a Bray Curtis dissimilarity (hereafter BC-dissimilarity) matrix using PRD-inducing capacity of soil as a factor.

### Total DNA extraction

For assessments of soil microbial communities, 5 to 10 g of the frozen soil samples were ground into powder using an MM 200 Mixer Mill homogenizer (Retsch, Newton, PA, USA). Total DNA was extracted separately from each sampling location within a managed block or zone; the MoBio PowerSoil Pro DNA isolation kit (MoBio Laboratories, Inc., Carlsbad, CA, USA) was used with 0.2 g of soil. The extracted DNA was further purified with MoBio PowerClean Pro kit (MoBio Laboratories, Inc., Carlsbad, CA, USA). DNA quality and yield were determined using the Nanodrop 2000 UV-Vis spectrophotometer (Thermo Fisher Scientific Inc., Waltham, MA, USA) and Qubit DNA ds HS assay system (Thermo Fisher Scientific Inc., Waltham, MA, USA), respectively.

### PCR amplification and illumina sequencing

A two-step PCR approach was used with multiple rRNA gene targets to integrate PCR amplification with addition of sample barcodes. Primers 515F/806R [21] and 799F/1193R [22] were used to target V4 and V5-V7 regions, respectively, of the bacterial 16S rRNA gene; primers ITS1f/ITS2 and fITS7/ITS4 were used for amplification of fungal ITS1 and ITS2 regions, respectively [23]; and primers ITS1oo/ITS7 were used for the amplification of oomycete ITS1 region (S1 Table) [24]. Each of these primers was tagged with a universal sequence (different for the forward and the reverse) to facilitate the addition of barcodes in the second PCR.

The two-step PCR approach involved dual indexed primer sets (S1 Table) [25]. The first PCR reaction mixture (25 μL) contained 1x buffer, 0.2mM deoxynucleotide triphosphates (dNTPs), 2.5 mM MgCl_2_, 2 μM tagged forward primer, 2 μM tagged reverse primer (Integrated DNA Technologies, Inc, USA), 3% dimethyl sulfoxide (DMSO) (Sigma Aldrich, USA), 0.5 U KAPA2G HotStart DNA Polymerase (KAPA Biosystems, Woburn, MA, USA) and 20–30 ng template DNA. The first PCR for bacteria included 15 cycles of previously described reaction parameters for the respective primer sets [21, 22], whereas the first PCR for fungi and oomycetes included 20 cycles of the published parameters [23, 24, 26] with a modification of increasing denaturation and polymerase activation temperature from 94 to 95°C. The second PCR was completed using reagents described for the first PCR, except that the template consisted of 1 μL of product from the first PCR mixture and the primers used targeted the universal sequence added in the first PCR. The primers in the second PCR also contained unique barcodes to facilitate demultiplexing the samples during sequence processing. Cycling parameters for the second PCR included an initial template denaturation and polymerase activation period of 1 min at 95°C, followed by 10 cycles of: 30 sec denaturation at 95°C, 30 sec of annealing at 60°C, 1 min of extension at 68°C, and 5 min final extension at 68°C.

For each primer set, a 5 μL aliquot of each final PCR product was examined via gel electrophoresis. PCR products were compared for band brightness, relative to 1kb Plus ladder (Thermo Fisher Scientific, Waltham, MA, USA) with ImageJ [27], then pooled to achieve similar concentrations prior to PCR purification as described previously in [28]. The libraries were purified using 0.8x Agencourt AMPure magnetic beads (Beckman Coulter, Sacramento, CA, USA). Amplicon sequencing was performed using Illumina MiSeq (v2 reaction kit) (2 × 300 bp paired-end) by the DNA Technologies Core Facility at University of California Davis, USA.

### Raw sequence data processing and bioinformatics pipelines

Raw paired-end FASTQ files were demultiplexed with the demux plugin (https://github.com/qiime2/q2-demux), and then primer sequences were removed with cutadapt plugin [29] in QIIME2 version 2019.4.0 [30]. Sequences were quality filtered, trimmed, denoised, and merged using the DADA2 algorithm [31]. Chimeric sequences were identified and removed during this step. The taxonomic affiliation for each bacterial amplicon sequence variant (ASV) was obtained using the Ribosomal Database Project (RDP) naive Bayesian rRNA classifier [32] against Silva v132 [33]. All reads assigned to chloroplast and mitochondria were removed from libraries before further analysis. A total of 1,350,129 and 421,504 reads remained for the V4 and V5-V7 bacterial libraries, respectively. For fungal sequencing data, Basic Local Alignment Search Tool (BLAST) was used to assign all sequence variants against UNITE + INSD (v6_sh_97) [34]. Sequences that were not classified to a fungal phylum were then compared with ITS fungal sequences in NCBI nr database using the BLAST [35]. The fungal data sets consisted of 935,315 and 732,671 reads for ITS1 and ITS2, respectively. For the oomycete amplicons, BLAST was used to assign all sequence variants against the NCBI nr database. For oomycete ITS1, a total of 1,128,832 reads were obtained. Reads that belonged to non-fungal or non-oomycete taxa were removed before further analyses of the data sets.

### Microbial community diversity and statistical analyses

PRIMER7, and the phyloseq and vegan packages in R, were used to analyze the microbial community diversity and composition at the ASV level [20, 36, 37]. Diversity estimates of observed ASVs, Shannon diversity index, and Pielou’s evenness were calculated using the vegan R package [37]. The statistical significance of differences in diversity estimates in PRD-inducing vs. non-inducing soils was tested by a non-parametric (ranked-based) Kruskal Wallis test. Relative abundances of different taxonomic groups were calculated using phyloseq; bar plots of the abundances were generated at the class and genus level for bacteria and fungi and at the genus level for oomycetes. Spearman’s correlation coefficients were determined between the class relative abundances and selected normalized soil physicochemical variables.

A cumulative sum scaling (CSS) normalization was performed on raw ASV tables to generate normalized counts using metagenomeSeq in R version 3.10 [38, 39]. CSS normalization is an extension of the quantile normalization approach suitable for marker gene surveys and helps to mitigate the influence of larger abundance values. We averaged the CSS-normalized microbial community replicates (n = 3 or 4) for each soil. This was necessary as the physicochemical and nematode data were measured from composites of the soil sampled in each block or zone as detailed above (obtained by combining the replicates rather than for each individual soil sample). These averaged data were imported into PRIMER7 for further analysis. To measure the structural diversity of bacterial, fungal, and oomycete communities, BC-dissimilarity matrices were constructed using the mean CSS-normalized and square-root-transformed data and visualized through non-metric multidimensional scaling (NMDS), in some cases with overlays of vectors for selected soil variables. The BC-dissimilarity matrices were examined for homogeneity of variance among PRD-inducing and non-inducing soils through permutation dispersion (PERMDISP) using deviations from centroid. Differences in the structure of microbial communities in PRD-inducing vs. non-inducing soils were assessed by subjecting the BC-dissimilarity matrices to PERMANOVA using 9999 permutations [40]. Hierarchical cluster analysis was performed among soils using BC-dissimilarity matrices. In order to compare the BC-dissimilarity matrices resulting from the complementary PCR primer sets as well as with Euclidean distances calculated for the soils based on selected soil physicochemical variables, the RELATE test was used with 9999 permutations. Also, the BEST Bio-Env analysis (selected BIOENV, which examines all possible combinations of physicochemical variables) in PRIMER7 was used to test relationships between community composition and physicochemical variables [20].

### Random forest modeling

Finally, to assess the overall value of soil physicochemical and microbial features for discriminating between PRD-inducing and non-inducing soils, we utilized random forest (RF) modeling approaches [41], implemented through R using the “randomForest” package version 4.6–14 [42]. Results of the PRD induction bioassay, classifying soils as PRD-inducing or non-inducing, were used as the qualitative response in RF classification. The control proportion for plant top weight from the greenhouse bioassay was used as the quantitative response variable for RF regression. We combined the CSS normalized, filtered and square root transformed microbial features from the bacterial V4 amplicons and fungal and oomycete ITS1 amplicons as well as selected physicochemical variables (percent clay, percent sand, pH, EC, total percent N, soluble Mg, and exchangeable-K) for the final RF analysis (total of 4457 features). We used 1001 trees in parameter “ntree”. The function “Importance” was used to measure the significance of each variable as the mean decrease in accuracy for RF classification, while the percent increase in mean squared error was used to assess variables for RF regression [41]. The model significance and cross-validation accuracy were determined using “rfUtilities” and “caret”, respectively, for RF classification and RF regression [43, 44].

## Results

### Prunus replant disease induction

**Fig 1:**
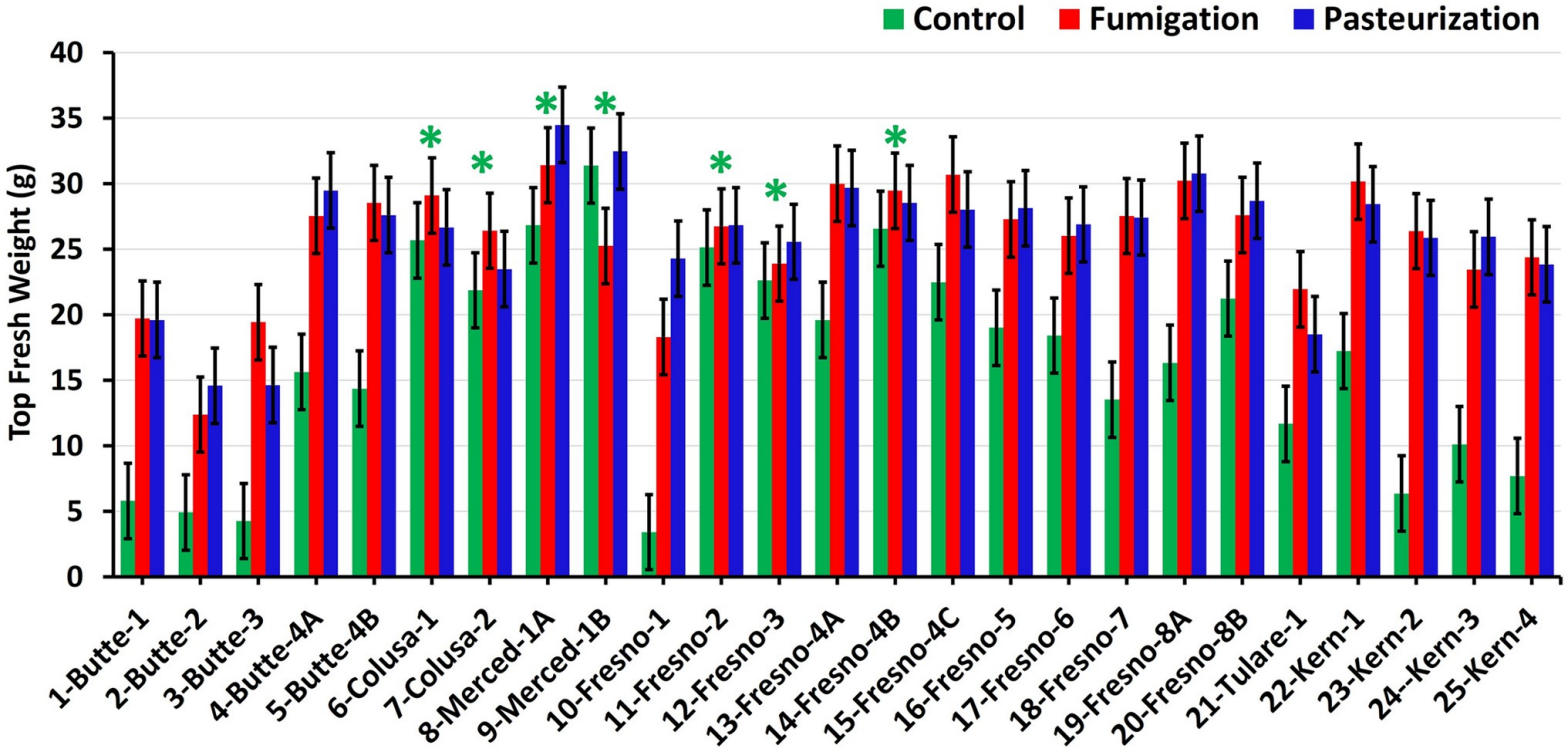
Final top fresh weight of Nemaguard peach seedlings as a function of soil source and preplant soil treatment in greenhouse bioassay. Labels on *x* axis indicate soil number and county-site, as in Table 1. Error bars are 95% confidence intervals. Asterisks indicate the non-inducing soils.

In the greenhouse bioassay, plant top and total fresh weights were affected by significant interaction between soil number (hereafter soil no.) and preplant soil treatment (Fig 1 and S1 Fig; *F* = 7.1, and 7.0, respectively, *P* <0.0001), indicating that the soils varied in PRD induction capacity. In any given soil, however, top and total fresh weights in fumigated and pasteurized treatments were statistically equivalent according to 95% confidence intervals, whether or not the remediation treatments stimulated plant growth (Fig 1 and S1 Fig). Top fresh weights were significantly increased by preplant soil fumigation and pasteurization in 18 of the 25 soils, which were considered PRD-inducing (Fig 1); results were similar with total plant fresh weights (S1 Fig). Top fresh weights were not significantly increased by preplant soil fumigation or pasteurization in the remaining seven soils, which were therefore considered non-inducing (Fig 1). All PRD-inducing soils originated from orchards that hosted a *Prunus* sp., whereas neither of the soils that hosted grape induced PRD. The field soils that had been fumigated before collection did not induce PRD, whereas the soil treated by ASD before collection was PRD-inducing.

### Physicochemical variables and nematode populations

**Fig 2:**
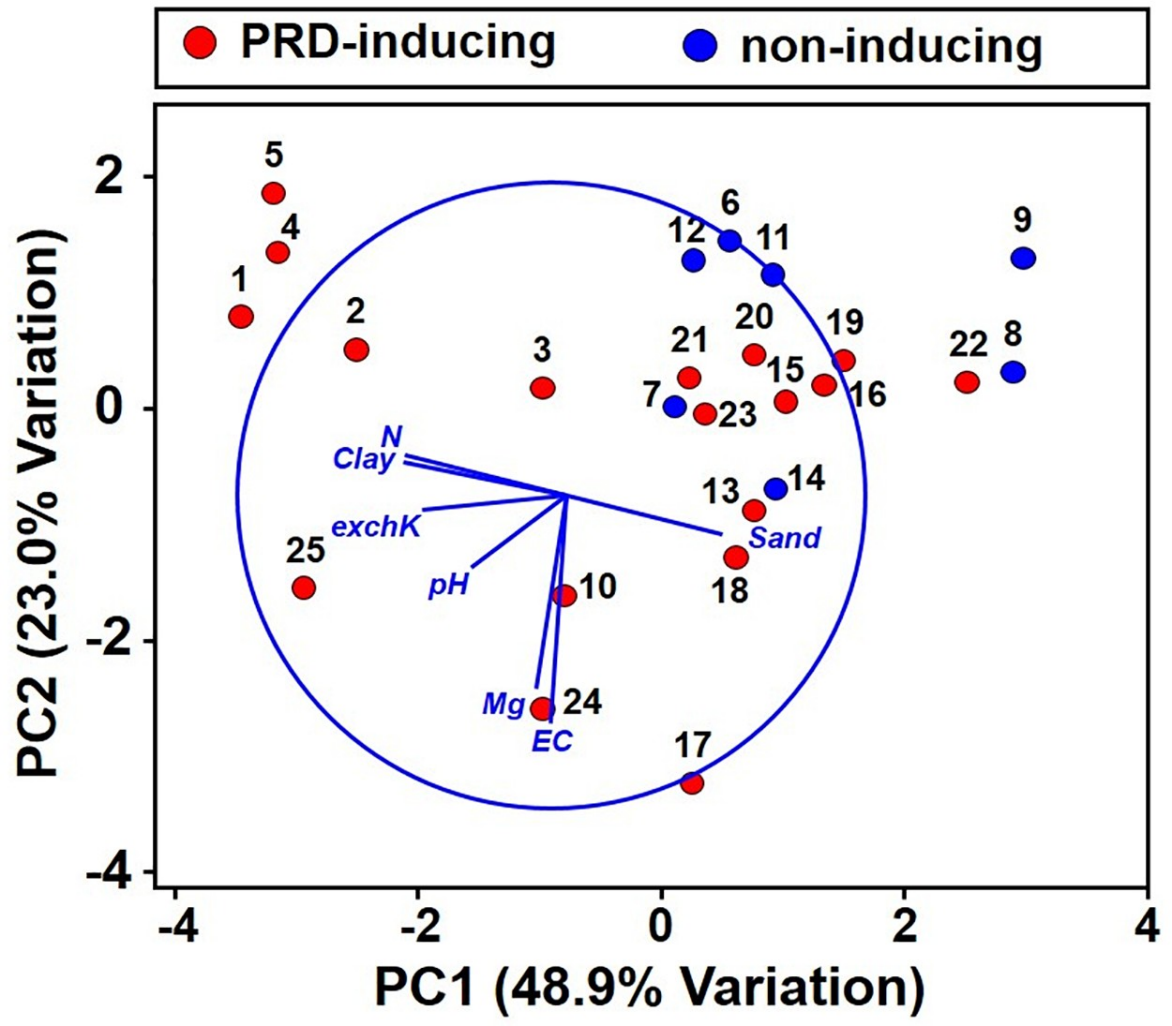
Principal component analysis of selected soil physicochemical variables among 25 soils. The numbers 1 to 25 correspond to soil numbers as listed in Table 1. Variables included were total % nitrogen (N); electrical conductivity (EC); exchangeable potassium (Exch-K); soluble magnesium (Mg), percent clay (clay), and percent sand (sand).

The physicochemical analyses among soils represented a wide range of texture classifications (clay loam to sand), pH (5.61 to 7.95), electrical conductivity (EC) values (0.5 to 3.3 dS/m) and soluble Mg (Table 1). In the PCA ordination of soils according to Euclidean distances among their normalized physicochemical variables, the first three principal components (PCs) explained most of the variation (85.9%), and PC1 and PC2 accounted for 48.9% and 23% of the variation, respectively (Fig 2). The first PC had a negative association with % clay, total % N and exchangeable-K and a positive association with % sand. The second axis had a large negative association with EC and soluble Mg. The PCA ordination distances seemed to reflect geographical sources more closely than PRD induction capacities; several PRD-inducing and non-inducing soils were ordinated in relatively close proximity to each other. Control proportions for top weight were negatively correlated with exchangeable-K (r = -0.73, *P* = <0.001), pH (r = -0.58, *P* = 0.003), % clay (r = -0.47, *P* = 0.02), total % N (r = -0.44, *P* = 0.03), and EC (r = -0.41, *P* = 0.04). The other soil physicochemical variables (% sand, % silt, and soluble Mg) were not significantly correlated with the control proportions for top weight (*P* > 0.05).

Several species of nematodes were identified among the 25 soils (Table 2). The pin nematode (*Paratylenchus* spp.) and free-living nematodes were among the most abundant. The ring nematode (*Mesocricinomella xenoplax*) was detected in relatively high counts in one of the grape soils (no. 11, Table 2) and one of the almond soils (no. 22) and in lower counts in several other soils (nos. 8, 9, 12, 16, 20). The lesion nematode (*Pratylenchus vulnus*), the root knot nematode (*Meloidogyne incognita*), and the dagger nematode were detected in several soils at relatively low counts but were absent from most of the soils (Table 2). There was no evidence for an association of nematode community composition with PRD; PERMANOVA of the BC-dissimilarity matrix for the complete set of counted nematode groupings revealed no significant difference between the PRD-inducing and non-inducing soils when soils in the same inducing category were considered replicates of one another (pseudo-F = 2.0769, *P* = 0.157).

**Table 2:** Nematode counts associated with the soils collected from 25 sites across northern, central, and southern portions of California’s Central Valley.

Soil no.	County-site	Common name of nematode species or species grouping and counts per 250 cc ^a^					
		Ring	Lesion	Root knot	Dagger	Pin	Free-living species
1	Butte-1	0	0	0	0	62	92
2	Butte-2	0	0	0	2	112	134
3	Butte-3	0	0	0	0	360	54
4	Butte-4A	0	0	0	0	104	8
5	Butte-4B	0	0	0	0	26	22
6	Colusa-1	0	0	0	0	646	64
7	Colusa-2	0	0	0	36	318	6
8	Merced-1A	30	0	0	0	0	54
9	Merced-1B	14	0	0	0	0	132
10	Fresno-1	0	0	0	0	883	29
11	Fresno-2	808	0	15	7	317	149
12	Fresno-3	56	0	0	22	544	336
13	Fresno-4A	0	0	0	0	4	248
14	Fresno-4B	0	0	0	0	0	178
15	Fresno-4C	0	0	0	0	0	586
16	Fresno-5	37	4	0	0	900	35
17	Fresno-6	0	13	0	0	538	134
18	Fresno-7	0	38	0	0	45	70
19	Fresno-8A	0	0	0	0	186	146
20	Fresno-8B	29	0	0	1	941	80
21	Tulare-1	0	0	0	27	662	92
22	Kern-1	892	184	3	38	179	42
23	Kern-2	0	0	0	0	226	34
24	Kern-3	0	0	0	0	824	58
25	Kern-4	0	4	0	45	500	89

^a^ “Ring” indicates *Mesocricinema xenoplax*; “Lesion” indicates *Pratylechus* sp.; “Root knot” indicates *Meloidogyne incognita*; “Pin” indicates *Paratylenchus* sp. All nematodes were extracted by centrifugal flotation and identified by morphological examination.

### Bacterial communities

**Fig 3:**
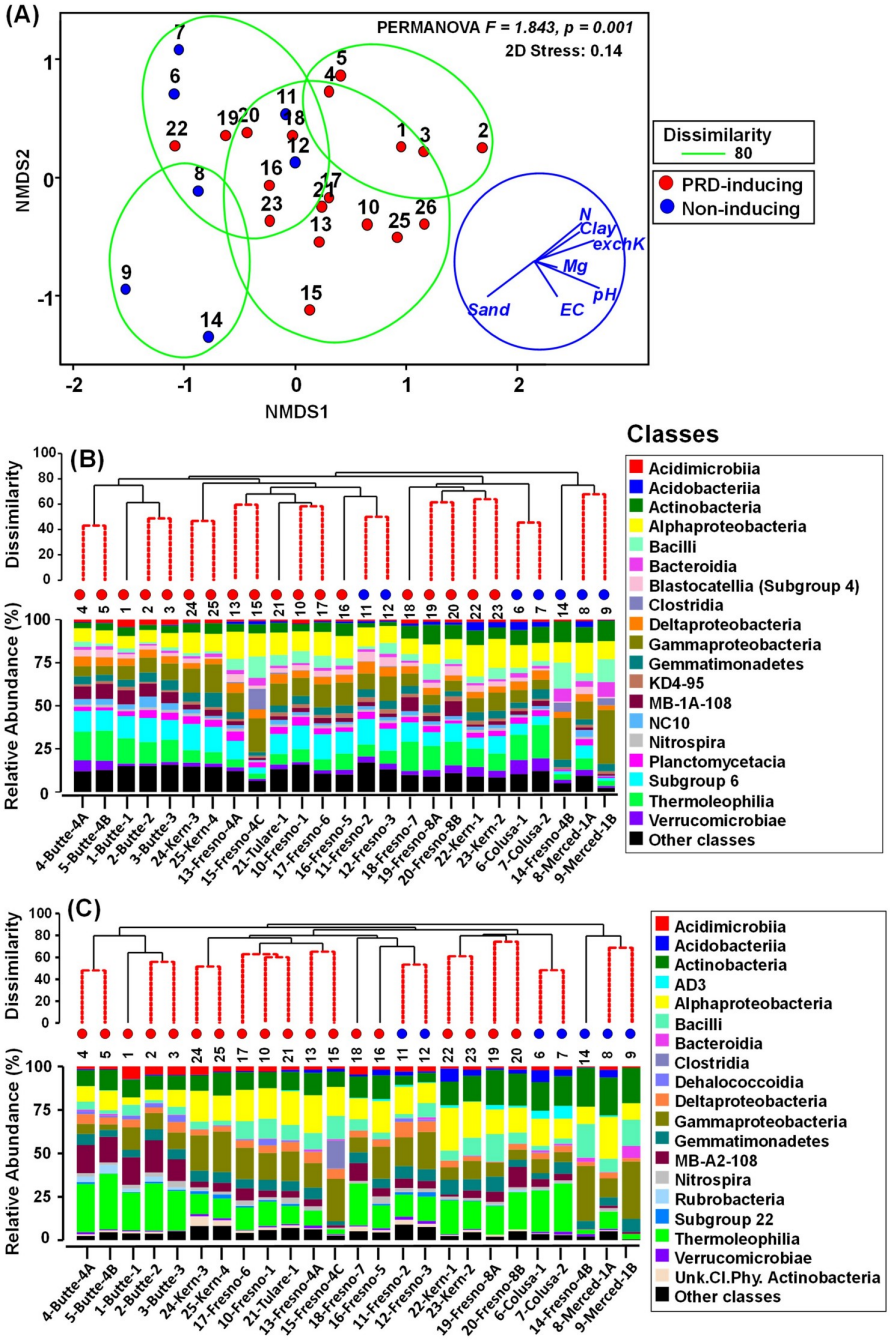
Characterization of soil bacterial community relationships and composition among PRD-inducing and non-inducing soils. **(A)** Non-metric multidimensional scaling (NMDS) ordination of soils based on Bray Curtis dissimilarity matrix (with overlays of hierarchical clustering at dissimilarity levels of 80% delimited by green lines and physicochemical vector relationships); **(B)** hierarchical clustering of soils based on BC-dissimilarity and class-level bacterial community composition of V4 amplicons; and **(C)** hierarchal clustering of soils based on BC-dissimilarity and class-level bacterial community composition of V5-V7 amplicons. Soils are identified by number or by number and county-site, as in Table 1. “Unk.Cl.Phy. Actinobacteria” represents ASVs classified only to phylum Actinobacteria. The BC-dissimilarities were based on cumulative sum scaling (CSS) normalized, square root-transformed counts of bacterial ASVs identified from rRNA gene amplicons in different soils.

PERMANOVA of BC-dissimilarity matrices at ASV level revealed significant differences in bacterial community composition between PRD-inducing and non-inducing soils with both amplicon sets (pseudo-F = 1.843 and *P* = 0.001 for V4; pseudo-F = 1.7072, *P* = 0.009 for V5-V7). Differences in dispersion were not significant between PRD-inducing and non-inducing soils for either amplicon set (PERMDISP, pseudo-F ≥ 0.811, *P* ≥ 0.896) using deviations from centroid. A RELATE test on BC-dissimilarities for the V4 vs. V5-V7 ASVs indicated close correspondence between community structures captured by each set of primer (*Rho* = 0.957, *P* = 0.0001, permutations = 9999); therefore ordination and hierarchal clustering results are presented only for the V4 amplicons. Despite the significance of PRD impacts on BC-dissimilarities according to PERMANOVA, NMDS ordination and hierarchal clustering of the soils according to their ASV-level BC-dissimilarities did not consistently discriminate between PRD-inducing and non-inducing soils (Fig 3A–3C). For instance, bacterial communities from two non-inducing grape soils (soil nos. 11 and 12) clustered closely with PRD-inducing nectarine soil (soil 16) (Fig 3A–3C).

There was a significant correlation between bacterial community BC-dissimilarities and soil physicochemical variable Euclidean distances (RELATE test; for V4 ASVs, *Rho* = 0.376, *P* = 0.001; for V5-V7 ASVs, *Rho* = 0.388, *P* = 0.001). Soil pH and exchangeable-K showed relatively high explanatory value for the bacterial community assemblage based on V4 ASVs (BEST Bio-Env test, *ρ*_*s*_ = 0.536, *P* = 0.001), while pH, exchangeable-K and percent sand were explanatory for assemblages based on V5-V7 ASVs (BEST Bio-Env test, *ρ*_*s*_ = 0.535, *P* = 0.001) (Table 3).

**Table 3:** Correlations between BC-dissimilarities and physicochemical variables as a function of PCR primer set^a^.

Community, primer set	Physicochemical variables with highest correlation to BC-dissimilarities	Spearman coefficient (*ρ*_*s*_)	Significance level, *P*
**Bacterial, V4 region**	pH and exchangeable-K	0.536	0.001
**Bacterial, V5V7 region**	Sand, pH and exchangeable-K	0.535	0.001
**Fungal, ITS1 region**	Silt, clay, pH and exchangeable-K	0.387	0.006
**Fungal, ITS2 region**	Silt, clay, pH and exchangeable-K	0.296	0.042
**Oomycete, ITS1 region**	Sand, pH, EC and exchangeable-K	0.57	0.001

^a^Spearman coefficients (*ρ*_s_) were calculated between transformed and normalized selected physicochemical variables (silt, clay, sand, pH, EC, total percent N, soluble Mg and exchangeable-K) and BC-dissimilarities of each primer using the BEST Bio-Env test in PRIMER7. Only the best combination of physicochemical variables with significant and highest Spearman coefficient values are included in table.

Among V4 amplicons, over 45% of the ASVs fell into four classes: Alphaproteobacteria (12.2%), Gammaproteobacteria (11.8%), Acidobacteria subgroup 6 (10.4%), and Thermoleophilia (9.52%) (Fig 3B). Among V5-V7 amplicons, 53% of the ASVs were in four classes: Alphaproteobacteria (14.7%), Gammaproteobacteria (14.4%), Thermoleophilia (16.3%), and Actinobacteria (12.9%), (Fig 3C). Relative abundances of certain taxa often varied between PRD-inducing and non-inducing soils. For example, class MB-A2-108 (phylum Actinobacteria) was more abundant (V4 ASVs 4.6%, V5-V7 ASVs 8.0%) in PRD-inducing soils compared to non-inducing soils (V4 ASVs 1.86%, V5-V7 ASVs 3.0%), while class Bacteroidia was more abundant in non-inducing soils (V4 ASVs 3.92%, V5-V7 ASVs 1.9%) compared to PRD-inducing soils (V4 ASVs 1.7%, V5-V7 ASVs 0.38%). Also, genus *Pseudomonas* was more abundant in non-inducing soils (V4 ASVs 9.7%, V5-V7 ASVs 9.6%) compared to in PRD-inducing soils (V4 ASVs 0.64%, V5-V7 ASVs 0.66%) S2A and S2B Fig). In some cases, abundances of certain taxa were correlated with physicochemical variables. For instance, the relative abundance of class Acidimicrobiia was positively correlated with levels of exchangeable-K (r = 0.72, for V4 and 0.49 for V5-V7 at *P* < 0.05), while abundances of Acidobacteria and Actinobacteria were negatively correlated with pH (for both taxa and both primer sets, r = -0.60 to -0.66; *P* < 0.05). Similar specific associations with physicochemical variables occurred with many other taxa (data not shown). The alpha diversity indices of richness (observed ASVs) and Shannon index values did not differ significantly between PRD-inducing and non-inducing soils (*P* > 0.05), but Pielou’s evenness indices were significantly higher in PRD-inducing soils than non-inducing soils (*P* = 0.043, for V4 only) (S2 Table).

### Fungal communities

**Fig 4:**
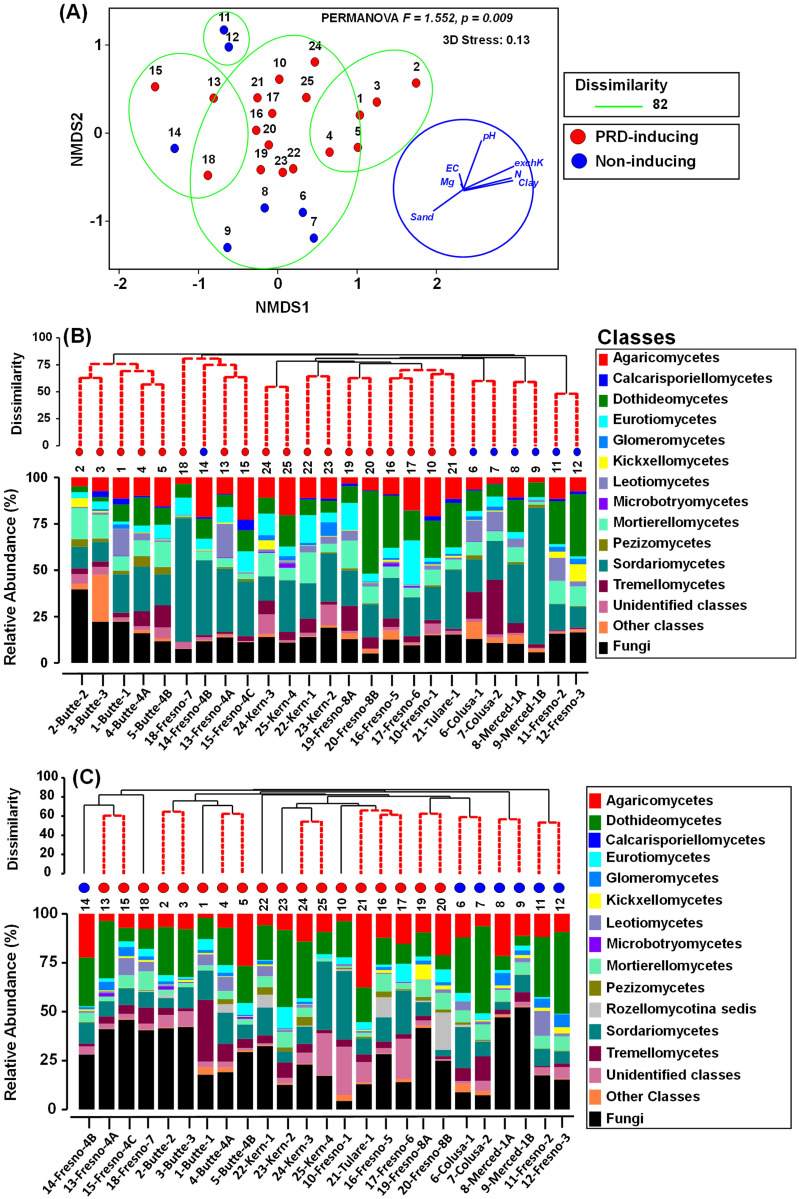
Characterization of soil fungal community relationships and composition among PRD-inducing and non-inducing soils. **(A)** Non-metric multidimensional scaling (NMDS) ordination of soils based on Bray Curtis dissimilarity matrix (with overlays of hierarchical clustering at dissimilarity levels of 82% delimited by green lines and physicochemical vector relationships); **(B)** hierarchical clustering of soils based on BC-dissimilarity and class-level fungal community composition of ITS1 amplicons; and **(C)** hierarchal clustering of soils based on BC-dissimilarity and class-level fungal community composition of ITS2 amplicons. Soils are identified by number or by number and county-site, as in Table 1. The BC-dissimilarities were based on cumulative sum scaling (CSS) normalized, square root- transformed counts of fungal ASVs identified from ITS regions amplicons in different soils.

PERMANOVA of BC-dissimilarity matrices revealed a significant difference in fungal community composition between PRD-inducing and non-inducing soils, both for ITS1 and ITS2 ASVs (pseudo-F = 1.552, *P* = 0.009 for ITS1; pseudo-F = 1.652, *P* = 0.007 for ITS2). Differences in dispersion were not significant between PRD-inducing and non-inducing soils for either amplicon locus (PERMDISP, pseudo-F = 1.686, *P* = 0.816 for ITS1; pseudo-F = 0.902, *P* = 0.814 for ITS2) using deviations from centroid. The RELATE test on BC-dissimilarities indicated close correspondence between community structures based on ITS1 vs. ITS2 ASVs (*Rho* = 0.851, *P* = 0.0001, permutation = 9999). Thus, we present NMDS ordination of soils based only on ITS1 BC-dissimilarities, and soils were not ordinated distinctly with respect to their PRD-inducing status (Fig 4A). Hierarchical cluster analysis using the ITS1-based ASVs BC-dissimilarity matrix showed five major clusters separated at 82% dissimilarity (Fig 4A, delimited by green lines), but the soils were not clustered consistently according to their PRD-inducing capacity; for instance, a non-inducing soil (Soil no. 14 from Fresno) with a history of fumigation was clustered with communities from PRD-inducing soils (soil nos. 13, and 15 from Fresno) Fig 4B and 4C).

There was a significant correlation between fungal community BC-dissimilarities and soil physicochemical Euclidean distances (RELATE test—ITS1 primers, *Rho* = 0.26, *P* <0.004; ITS2 primers, *Rho* = 0.191, *P* <0.031). Furthermore, a best combination of physiochemical variable was identified that were weakly but significantly correlated with the fungal community structure based on BC-dissimilarities using ITS1 and ITS2 amplicons (BEST Bio-Env test, *ρ*_*s*_ = 0.387, *P* = 0.006, for ITS1; *ρ*_*s*_ = 0.296, *P* = 0.042 for ITS2) (Table 3).

Many distinctions were possible among soil fungal communities resolved in ITS1 and ITS2 amplicons, yet few were consistent in relation to PRD induction potential. Among ITS1 amplicons, class Sordariomycetes was prevalent in all soils, and it dominated in several from Fresno County (66% in soil no. 14; 40.3% in no.13; 40.3% and 34.1% in no. 15) (Fig 4B). Also, class Tremellomycetes dominated in the non-inducing soil no. 7 (29.3%), while in other soils it was less than 14.5%. Among ITS2 amplicons, fungi that were resolved only to kingdom level dominated in all soils. At class level, Dothideomycetes were abundant in all non-inducing soils (24.9% to 44.5%) and three PRD-inducing soils (no. 2, 25%; no. 13, 29.3%; and no. 22, 39.4%) (Fig 4C). At genus level, *Trichoderma* was more abundant in non-inducing soils (ITS1 region, 14.8%, ITS2 region 13.8%) than in PRD-inducing soils (ITS1 region 4.2%, ITS2 region 1.8%) (S2C and S2D Fig). There were no consistent indications of important alpha diversity shifts between PRD-inducing and non-inducing soils (S2 Table).

### Oomycete communities

**Fig 5:**
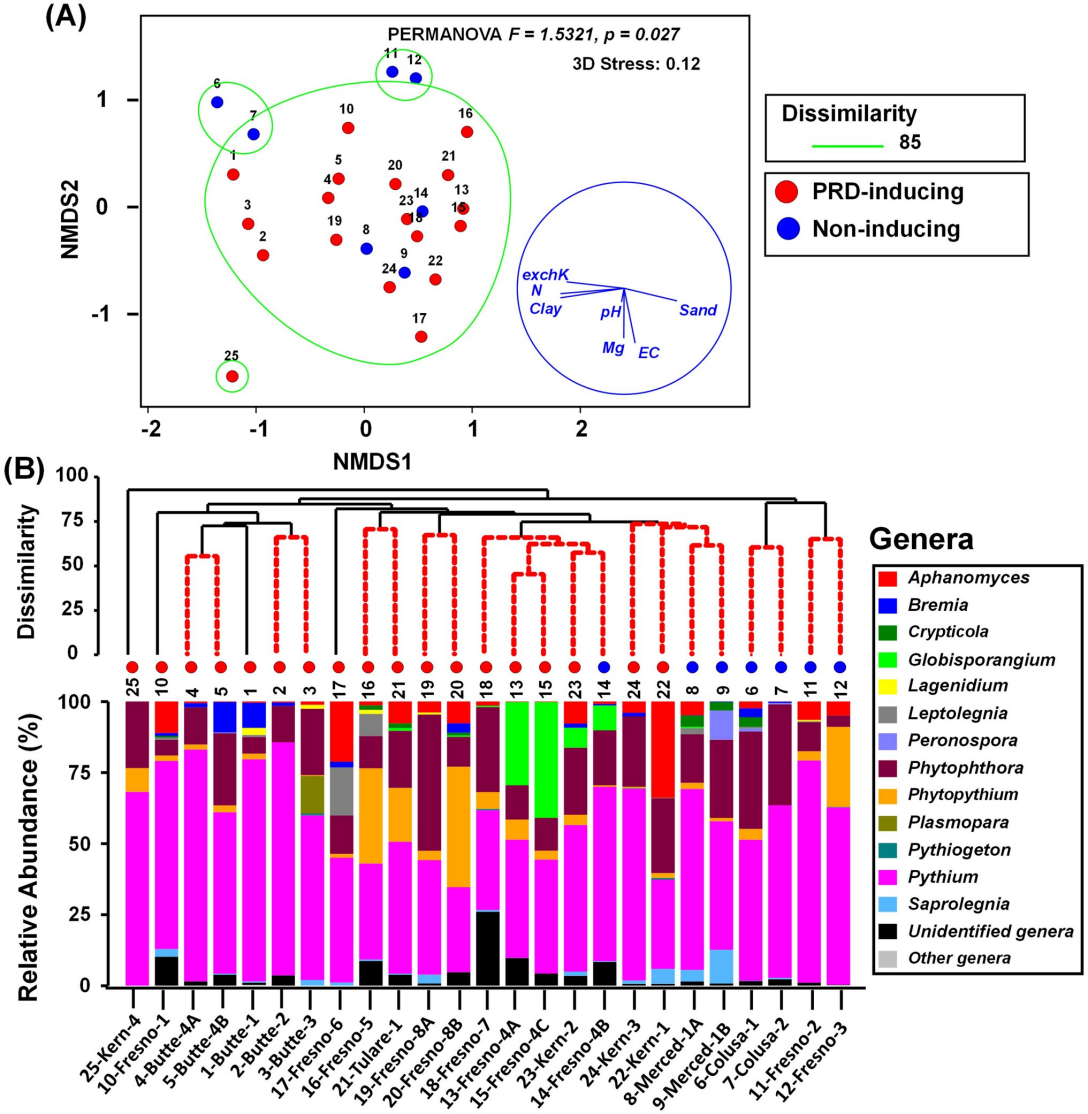
Characterization of soil oomycete community relationships and composition among PRD-inducing and non-inducing soils. **(A)** Non-metric multidimensional scaling (NMDS) ordination of soils based on Bray Curtis dissimilarity matrix (with overlays of hierarchical clustering at dissimilarity levels of 85% delimited by green lines and physicochemical vector relationships) and **(B)** hierarchical clustering of soils based on BC-dissimilarity and genus-level oomycete community composition of ITS1 amplicons. Soils are identified by number or by number and county-site, as in Table 1. The BC-dissimilarities were based on cumulative sum scaling (CSS) normalized, square root- transformed counts of oomycete ASVs identified from ITS1 region amplicons in different soils.

PERMANOVA of BC-dissimilarity matrices based on ITS1 ASVs indicated that there were significant differences in the oomycete communities harboured by PRD-inducing vs. non-inducing soils (pseudo-F = 1.5231 and *P* = 0.026). Differences in dispersion were not significant between PRD-inducing and non-inducing soils (PERMDISP, pseudo-F = 1.109, *P* = 0.676) using deviations from centroid. The NMDS ordination of soils based on the ITS1 BC-dissimilarity matrix did not consistently cluster PRD-inducing soils separately from non-inducing soils (Fig 5A). Hierarchical cluster analysis using the ITS1 BC-dissimilarity matrix showed four major clusters separated at 85% dissimilarity (Fig 5A, delimited by green lines), but the soils were not clustered consistently according to their PRD-inducing capacity (Fig 5A and 5B). A best combination of physicochemical variables (sand, pH, EC, and exchangeable-K) was identified and exhibited relatively high correlation with oomycete community BC-dissimilarities among soils (BEST Bio-Env test, *ρ*_*s*_ = 0.57, *P* = 0.001) (Table 3).

Among oomycete taxa comprising oomycete communities, *Pythium* was the most prevalent genus in all soils, accounting for 45 to 81% of the total relative abundance (Fig 5B). *Phytophthora*, the second most abundant genus, accounted for 4 to 48% of relative abundance. *Phytopythium* had high relative abundance in soil nos. 12 (28.2%), 20 (42.2%), and 21 (19%), compared to other soils (<8.4%) (Fig 5B). Between the PRD-inducing and non-inducing soils, *Pythium* had higher relative abundance in non-inducing (61.2%) than in PRD-inducing (53.5%) soils (S2E Fig). In some cases, specific oomycete taxa had a strong correlation to physicochemical variables; for instance, the relative abundance of *Pythium* was positively correlated with % clay, pH and exchangeable-K (r = 0.48 to 0.56, *P* < 0.05), whereas, the relative abundance of *Phytophthora* was negatively correlated with pH (r = -0.47, *P* < 0.05).

### Random forest modeling

**Fig 6:**
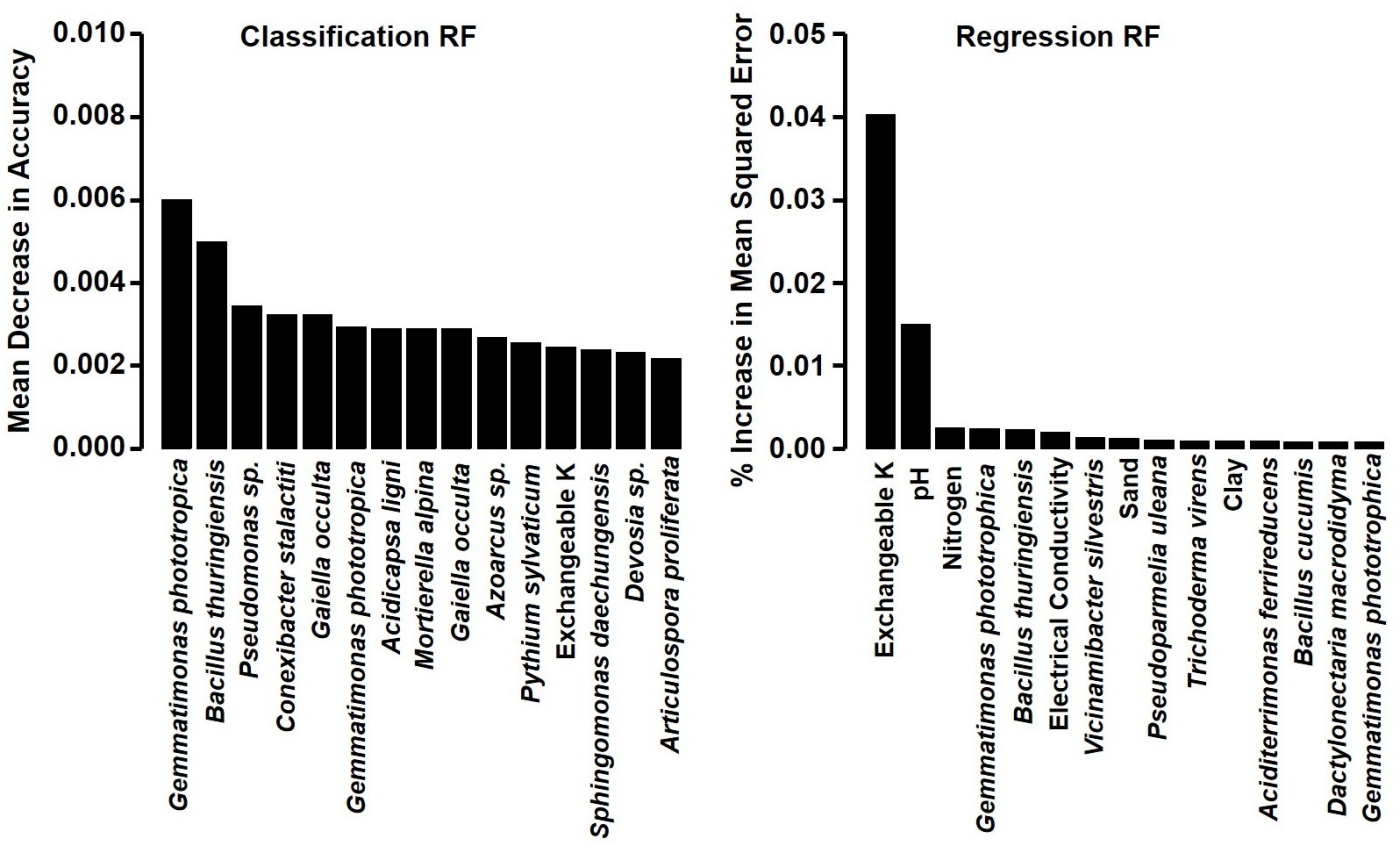
Top 15 most-important features in Random forest modeling for discrimination between PRD-inducing and non-inducing soils. In the RF-classification model, variable importance was indicated and listed in order by the mean decrease in accuracy. In the RF-regression model, variable importance was indicated and listed in order by percent increase in mean squared error. Performance and accuracy were significant in each model (kappa value of 0.92 for classification model and low mean absolute error of 0.083 for regression model).

Based on the microbial features of bacterial, fungal, and oomycete ASV abundances (which PERMANOVA had indicated contained structure related to PRD-induction potential), and soil physicochemical features of percent clay, percent sand, percent silt, pH and exchangeable-K (which BEST Bio-Env analyses revealed to relate significantly to structure of the microbial communities), a RF model classified samples effectively with an out-of-bag (OOB) error rate of 1.08%. The RF classification model performance was further assessed by “leave-one-out” cross-validation, showing accuracy of 96% (kappa value of 0.92) in classifying soils correctly into PRD-inducing and non-inducing categories. Among the top 15 most important features based on RF-classification, certain bacterial ASV abundances were the variables most commonly selected and had the greatest explanatory value (Fig 6, S3 and S4 Tables). Interestingly, the RF-informative bacterial ASVs were more abundant in non-inducing soils than PRD-inducing soils (S3 Fig). Only two fungal ASVs and one oomycete ASV were among the top 15 features selected by RF-classification, and all three of these were more abundant in non-inducing than in PRD-inducing soils (S4 Fig). Among soil physicochemical variables, only exchangeable-K was among the top 15 features in RF-classification. The RF-regression model had a very low mean of squared residuals (MSE = 0.01), with high percentage of variation explained (84.3%). The model was assessed by “leave-one-out” cross-validation, and gave an R^2^ value of 87%. Based on RF-regression analysis, exchangeable-K and pH were the most important features with the highest percent increase in mean squared error values (Fig 6).

## Discussion

Our report identified multiple soil physicochemical and microbial variables in 25 diverse orchard and vineyard replant soils that collectively discriminated between soils that did or did not induce PRD in a greenhouse bioassay. It is logical to consider whether or not the greenhouse bioassay reflected PRD induction capacities under orchard conditions. Although the bioassay has not been rigorously validated against experimental field responses, there is evidence for useful correspondence between greenhouse bioassay and orchard trial responses. For example, the orchard supplying soil no. 6 did not respond significantly to preplant soil fumigation in the field, nor did peach seedlings respond significantly to fumigation or pasteurization of that soil in the bioassay. Conversely, orchards planted where soils 8 and 13 were collected responded positively to preplant soil fumigation, as did plants in the bioassay (note: in soil 8, PRD was induced according to plant total weights but not top weights, S1 Fig). Bioassay-field correspondences were also evident in soil nos. 9 and 14: these soils were collected from zones (plots) that had been field fumigated, and accordingly, PRD was not induced by them in the bioassay. Also, grape vineyard soils do not typically induce immediate growth suppression in subsequent orchard plantings of *Prunus* (Browne, *unpublished*), nor did they induce PRD in the bioassays of soil nos. 11 and 12 from grape vineyards. Interestingly, however, PRD induction from soil no. 15 in the bioassay was not consistent with corresponding orchard data. Soils no. 13, 14, and 15 were collected, respectively, from the control, fumigated, and ASD-treated plots of a field trial. In the orchard, both the ASD and the fumigation treatments significantly and equally increased orchard tree growth and nut yields [4, 17], which is contrary to the PRD induction shown by soil no. 15 in the bioassay. Although reasons for this bioassay-orchard discrepancy are unknown, it is possible that mechanisms of fumigation and ASD differ in ways not accounted for adequately in the bioassay. Clearly, further orchard validation of the PRD bioassay is advisable.

Crop history and preplant soil treatments in the field seemed to account for much, but not all, of the capacity for PRD induction among soils. All of the 18 soils that were PRD-conducive in the bioassay had recently hosted a *Prunus* spp., while neither of the two soils from grape vineyards induced PRD. Of the remaining five soils that had hosted *Prunus* yet were not conducive to PRD, two had been field fumigated, which typically prevents PRD. The other three soils—nos. 6 and 7 (Colusa-1and Colusa-2, respectively) and 8 (Merced-1A) had not been field fumigated and still did not induce PRD in the bioassay, although soil no. 8 induced PRD in terms of total plant fresh weights (S1 Fig). Soils 6 and 7 from Colusa County were lowest in pH (pH 5.6–5.8), and our PCA and correlation analyses of physicochemical variables indicated that soil pH at the time of soil collection was negatively correlated with the control proportion of plant top fresh weight among the 25 soils in the bioassay. Conditions of the bioassay, including the use of Hoagland’s solution and addition of sterile sand, altered some or all the variables from their conditions in the original soils at the time of collection, but it seems likely that initial values of these variables significantly shaped PRD induction capacity of the soils.

Among the soils examined, there was no evidence for a relation between nematode community structure and capacity for PRD induction. With respect to counts of the pin nematode (*Paratylenchus* sp.) and free-living nematode species, the lack of correspondence between abundances and PRD induction was not surprising; *Paratylenchus haematus*, the most common species of pin nematode found on perennial crops in California, can feed and reproduce on *Prunus* but is not known to affect tree vigor or yield [45], and counts of the free-living group of non-phytopathogenic nematodes would only be expected to have an indirect relationship to plant growth [46]. Also, it is uncertain whether the duration of the plant bioassay trial was sufficiently long to permit nematodes to impact plant growth; in orchards, nematode populations can take several years to build after the soil disturbances involved in orchard removal and replanting [47]. Thus, the bioassay was more appropriate for testing PRD mediated by microbial consortia than for testing for growth suppressing potential of PPN in the soil. Our bioassay results were consistent with current conceptions of PRD and nematode parasitism as two distinct replant problems, the former more immediate in impact and the latter more of a long-term threat, but both affecting cultivated species of *Prunus*.

We used complementary primer sets targeting different regions of the rRNA gene for bacteria and fungi. Primer selection is a critical consideration in amplicon-based community analysis, for several reasons, including differences in amplification bias and resolution among taxa [48, 49]. In this study, bacterial amplicons from the V4 region resolved many more ASVs (6,517) than those from the V5-V7 region (2,166). Although the amplicons from V4 and V5-V7 supported similar conclusions in our study, it is possible the improved resolution from V4 could be valuable under some circumstances. Among fungi, our ITS1 amplicons afforded better taxonomic resolution than our ITS2 amplicons; a smaller proportion of ASVs identified only to kingdom level resulted from ITS1 (17%) than from ITS2 (32%). Indeed, ITS1 has been recommended for universal fungal barcoding over ITS2 due to the former’s superior taxonomic resolving power and consistency of ITS1 amplicon results with metagenomic shotgun sequencing results [50, 51]. Our comparisons suggest that, in future examinations of bacterial and fungal communities in California replant soils, V4 and ITS1 amplicons would be favored over V5-V7 and ITS2 amplicons.

The significance of pH and exchangeable-K stood out in our examinations; they both correlated negatively with control top weight proportions (i.e., indicating more severe disease as either exchangeable-K or pH increased), and they both related significantly to the structure of bacterial, fungal, and oomycete communities in the 25 soils. They were the only measured variables exhibiting consistent significance in relation to each microbial community. There are many reports, both from agricultural and natural ecosystems, of significant relationships between soil pH and communities of bacteria [52, 53], fungi [53, 54], and oomycetes [55]. Fewer reports are available on relationships between exchangeable-K and these communities [56]. Regarding our work, knowledge of relationships among PRD with soil physicochemical and microbial variables could be useful for predicting the need for soil remediation by fumigation, ASD, or agronomic approaches that may suppress the disease. As an example of the potential for applications of such knowledge, incidence and severity of potato scab caused by *Streptomyces scabies* can be suppressed in many soils by reducing pH to ≤5.5 [57]. Of course, the nature of pH and exchangeable-K relations to PRD are unknown and will require further exploration to determine if agronomic manipulations of them would be effective. California soils do vary widely in pH, exchangeable-K, and other correlated physicochemical variables subject to agronomic adjustments, indicating value in further study of them in relation to PRD.

Ultimately, we used RF modeling to identify physicochemical and microbial variables that best discriminated between non-inducing and PRD-inducing soils. Among the 15 most important discriminating ASV abundance variables identified in our RF classification modeling, 11 were bacterial, two were fungal and one was oomycete-based, and all had higher relative abundances in non-inducing soils than in PRD-inducing soils. Exchangeable-K was the only physicochemical variable among the top 15 RF classification features. Among genera and species represented among the RF-informative ASV variables, detailed descriptions involving agricultural soils are only available in the literature for *Bacillus thuringensis*, *Pseudomonas* sp., *Azoarcus* sp., *Mortierella alpina*, and *Pythium sylvaticum*. *Bacillus thuringeniesis* is a well-known soil inhabitant that has been used as a biocontrol agent against many pests, including oomycetes and insects [58, 59]. Relatedly, OTUs of *Bacillus* spp. were more prominent among healthy rhizosphere soil samples than in corresponding samples impacted by apple replant disease [13]. Many species of *Pseudomonas* can suppress soilborne plant pathogens [60, 61], but previous work reported that abundance of *Pseudomonas* OTUs correlated negatively with peach shoot weights [16]. Species of *Mortierella*, including *M*. *alpina*, are common in soil as saprophytes [62, 63]. *Azoarcus* sp. can fix nitrogen and has been reported as a root endophyte [64]; however, in contrast to our results, *Azoarcus* and *Gaiella* were negatively correlated with peach seedling growth [16]. *Pythium sylvaticum* (also known as *Globisporangium sylvaticum*) is a documented plant pathogen, but not on peach [65, 66], nor did we find evidence of peach pathogenicity. Several of the ASVs identified as top predictors in RF classification are known as degraders of contaminants in soil or water [64, 67], which may have significance for soil health and plant growth. Our results, with the discriminating ASVs occurring in greater abundance in non-inducing soils, indicate that concepts of PRD induction should not only consider potential impacts of soilborne pathogens, but assess impacts of microorganisms that may suppress pathogens or stimulate plant growth. Although the microbial variables were the most discriminating features in RF classification of soils as PRD inducing or non-inducing, environmental variables (especially exchangeable-K and pH) were prominent for RF regression (i.e., which assessed relations of variables to PRD severity), emphasizing the importance of both types of variables for continuing examinations of PRD induction.

## Conclusion

A greenhouse bioassay provided a useful foundation for examining relationships between soils’ capacities to induce PRD and key soil physicochemical and microbial community variables. Among the soils examined, exchangeable-K and pH levels were significantly correlated with structure of bacterial, fungal, and oomycete communities in the soils, as well as incidence and severity of PRD induction. RF-classification modeling confirmed the importance of multiple physicochemical and microbial community variables in discriminating between PRD-inducing and non-inducing soils, and RF-regression modeling confirmed the value of physicochemical and microbial variables in modeling PRD severity. We posit that examinations of root microbial communities associated with PRD and soil physicochemical and microbial community variables would be informative and complement this work. Rigorous validations of the PRD bioassay against orchard results, including assessments of *in situ* soil environmental and microbial variables, could prove valuable and are underway.

## Supporting information

S1 Fig Final mean total fresh weight of Nemaguard peach seedlings as a function of soil source and preplant soil treatment in greenhouse bioassay. Labels on *x* axis indicate soil number and county-site, as in Table 1. Error bars are 95% confidence intervals. Asterisks indicate the non-inducing soils.
(TIF)

S2 Fig Relative abundances in bacterial, fungal and oomycete communities associated with PRD-inducing and non-inducing soils. **(A and B)** Relative abundances of top 19 most abundant genera of bacterial community based on V4 and V5-V7 amplicons, respectively. **(C and D)** Relative abundances of top 20 most abundant genera of fungal community based on ITS1 and ITS2 amplicons, respectively. **(E)** Relative abundances oomycete genera based on ITS1 amplicons.
(TIF)

S3 Fig Box plots of relative abundances in non-inducing and PRD-inducing soils among informative bacterial ASVs identified by RF modeling. Blue shading represents abundances in non-inducing soils, and red shading represents abundances in PRD-inducing soils.
(TIF)

S4 Fig Box plots of relative abundances in PRD-inducing and non-inducing soils of informative fungal and oomycete ASVs identified by RF modeling. Blue shading represents abundances in non-inducing soils, and red shading represents abundances in PRD-inducing soils.
(TIF)

S1 Table Primers used in this study to amplify marker genes for bacterial, fungal and oomycete communities. (DOCX)

S2 Table Diversity estimates for microbial communities associated with PRD-inducing and non-inducing soils. (DOCX)

S3 Table NCBI Blast results for top-ranked ASVs based on RF classification of bacterial, fungal and oomycete features. (DOCX)

S4 Table NCBI Blast results for top-ranked ASVs based on RF regression of bacterial, and fungal features. (DOCX)

S1 File Soil physicochemical properties and plant growth data. (XLSX)
