# Supplementary figures and images for: Characterization of soils conducive and non-conducive to Prunus replant disease
Source: PLoS One. 2021 Dec 10;16(12):e0260394. doi: 10.1371/journal.pone.0260394 (PMC8664177; doi:10.1371/journal.pone.0260394)

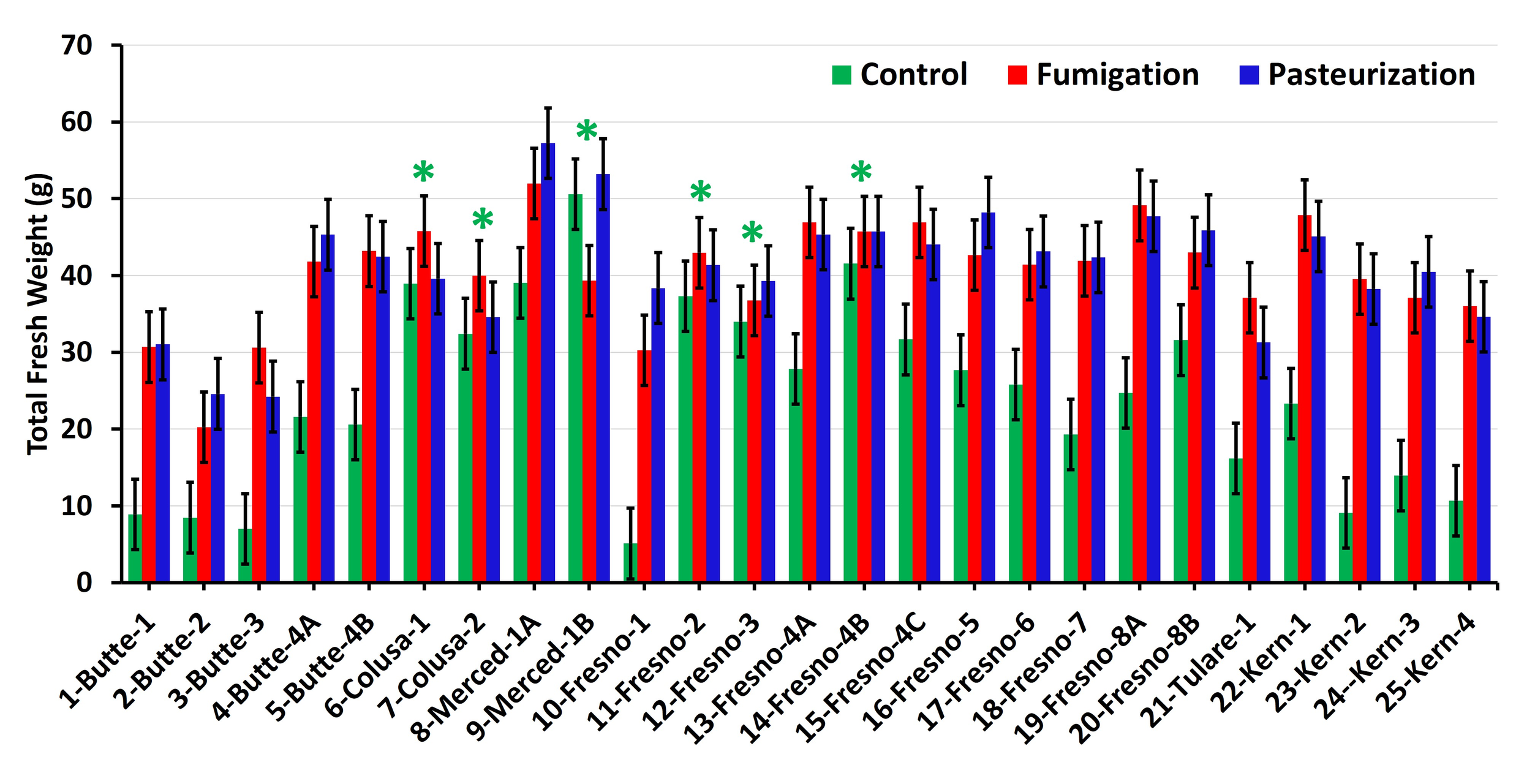

Supplement: S1 Fig — Labels on x axis indicate soil number and county-site, as in Table 1. Error bars are 95% confidence intervals. Asterisks indicate the non-inducing soils. (TIF) [file pone.0260394.s001.tif]

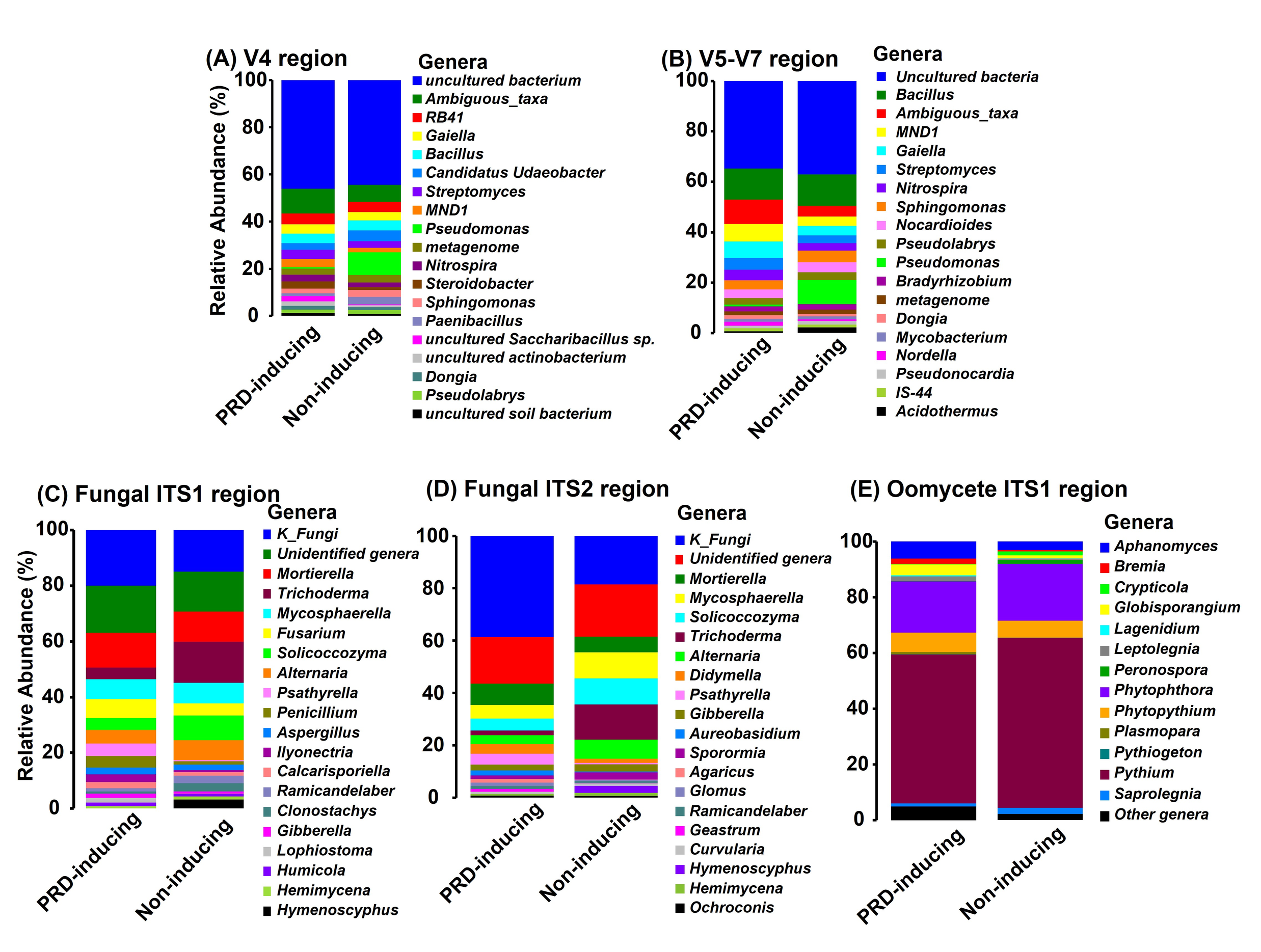

Supplement: S2 Fig — (A and B) Relative abundances of top 19 most abundant genera of bacterial community based on V4 and V5-V7 amplicons, respectively. (C and D) Relative abundances of top 20 most abundant genera of fungal community based on ITS1 and ITS2 amplicons, respectively. (E) Relative abundances oomycete genera based on ITS1 amplicons. (TIF) [file pone.0260394.s002.tif]

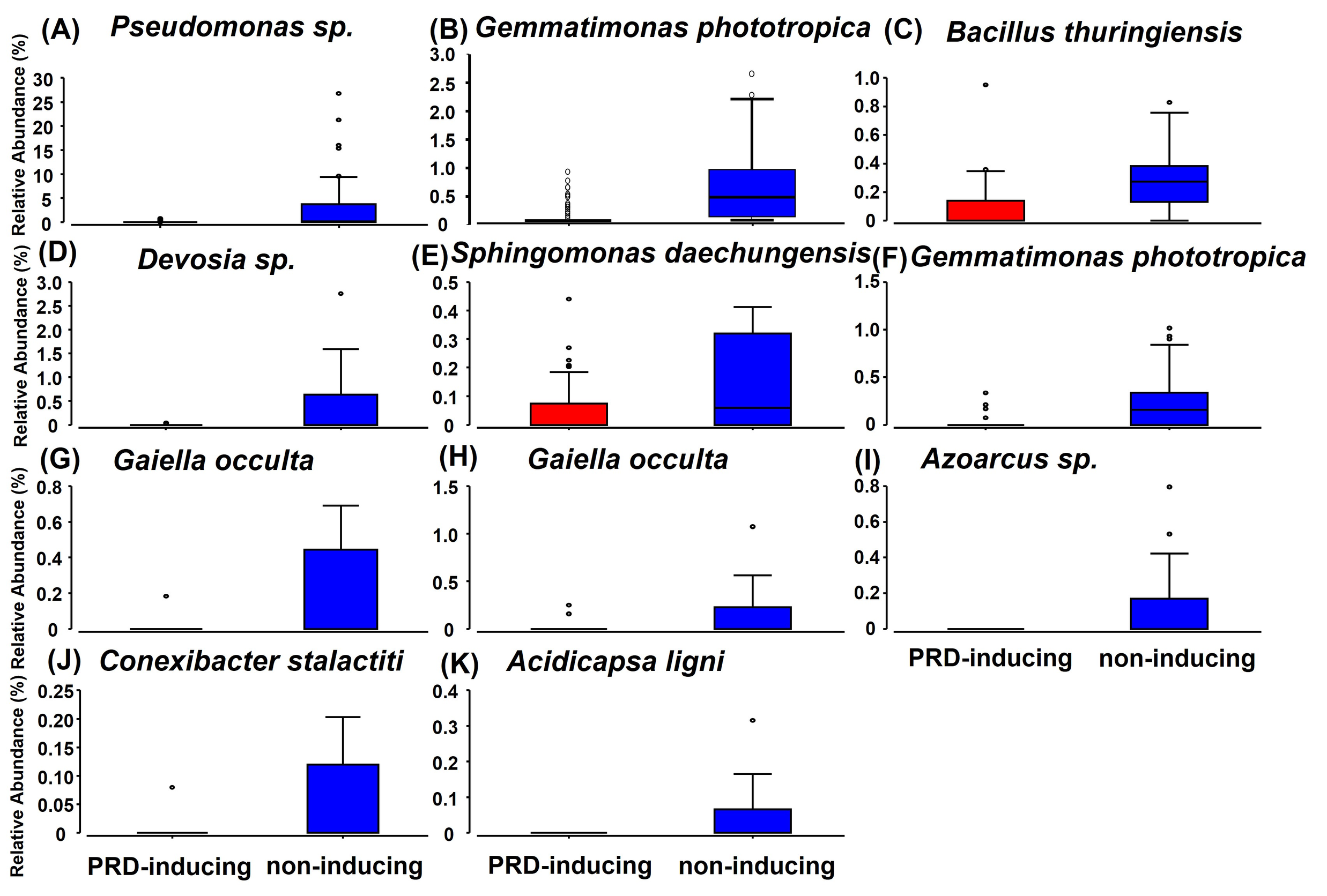

Supplement: S3 Fig — Blue shading represents abundances in non-inducing soils, and red shading represents abundances in PRD-inducing soils. (TIF) [file pone.0260394.s003.tif]

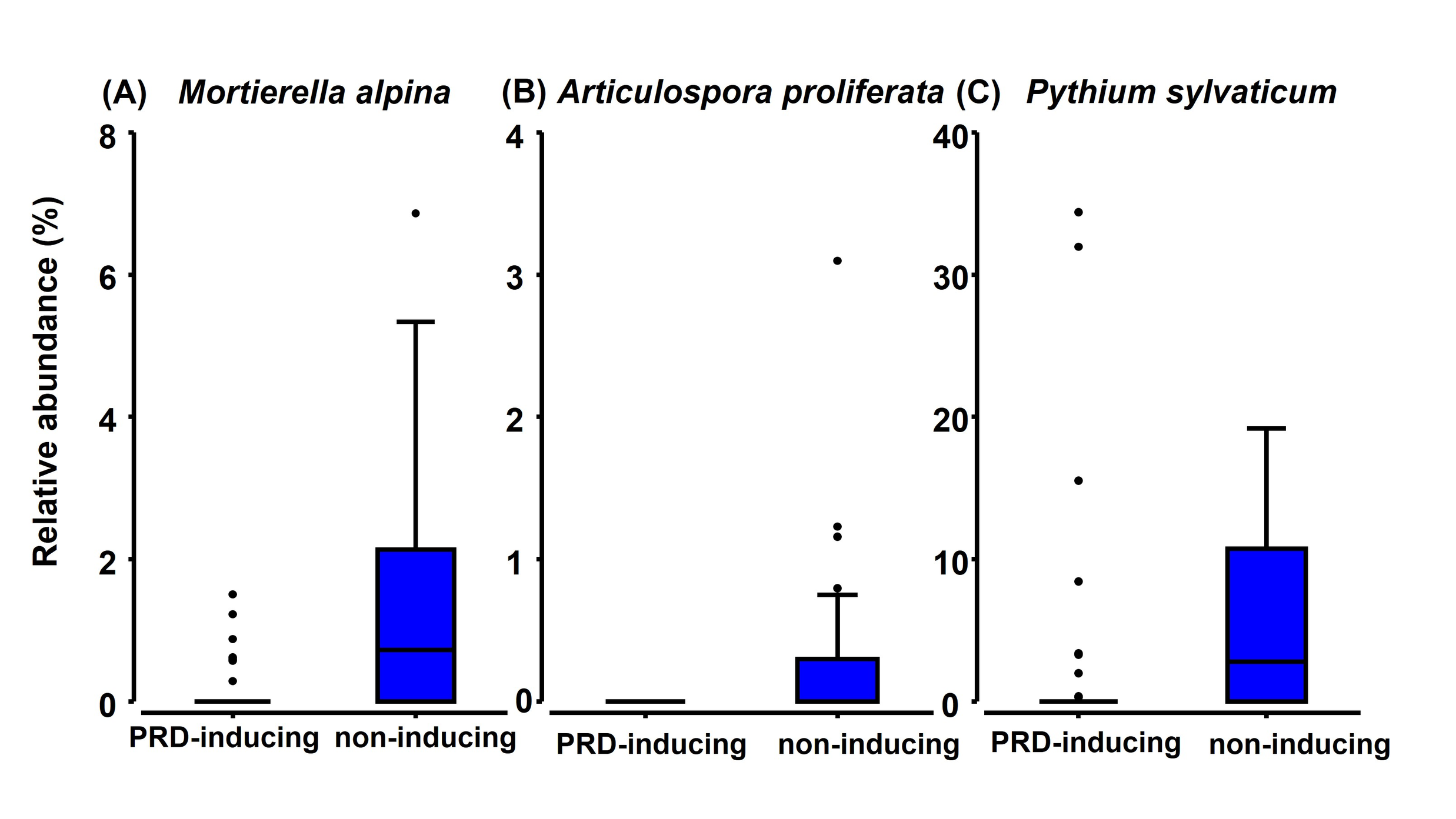

Supplement: S4 Fig — Blue shading represents abundances in non-inducing soils, and red shading represents abundances in PRD-inducing soils. (TIF) [file pone.0260394.s004.tif]
