# Supplementary material for: Characterization of soils conducive and non-conducive to Prunus replant disease
Source: PLoS One. 2021 Dec 10;16(12):e0260394. doi: 10.1371/journal.pone.0260394 (PMC8664177; doi:10.1371/journal.pone.0260394)
Supplement: S1 Table — (DOCX) [file pone.0260394.s005.docx]

**S1 Table**. Primers used in this study to amplify marker genes for bacterial, fungal and oomycetes communities

| **Primer name** | **Tag sequence** | **Spacer sequence** | **Linker sequence** | **Target-specific sequence** |
| --- | --- | --- | --- | --- |
| **515F** | TCGTCGGCAGCGTCAGATGTGTATAAGAGACAG | none or A or AT or ATC | GT | GTGYCAGCMGCCGCGGTAA |
| **806R** | GTCTCGTGGGCTCGGAGATGTGTATAAGAGACAG | none or A or AT or ATG | CC | GGACTACNVGGGTWTCTAAT |
| **799F** | TCGTCGGCAGCGTCAGATGTGTATAAGAGACAG | none or A or AT or ATC | GT | AACMGGATTAGATACCCKG |
| **1193R** | GTCTCGTGGGCTCGGAGATGTGTATAAGAGACAG | none or A or AT or ATC | GC | ACGTCATCCCCACCTTCC |
| **ITS1f** | TCGTCGGCAGCGTCAGATGTGTATAAGAGACAG | none or A or AT or ATC | GG | CTTGGTCATTTAGAGGAAGTAA |
| **ITS2** | GTCTCGTGGGCTCGGAGATGTGTATAAGAGACAG | none or A or AT or ATG | CG | GCTGCGTTCTTCATCGATGC |
| **fITS2** | TCGTCGGCAGCGTCAGATGTGTATAAGAGACAG | none or A or AT or ATC | GG | GTGARTCATCGAATCTTTG |
| **ITS4** | GTCTCGTGGGCTCGGAGATGTGTATAAGAGACAG | none or A or AT or ATG | CG | CCTCCGCTTATTGATATGC |
| **ITS1oo** | TCGTCGGCAGCGTCAGATGTGTATAAGAGACAG | none or A or AT or ATC | AA | GGAAGGATCATTACCACA |
| **ITS7** | GTCTCGTGGGCTCGGAGATGTGTATAAGAGACAG | none or A or AT or ATG | CG | AGCGTTCTTCATCGATGTGC |

In the first PCR (step 1), target specific primers with “tails” at their 3’ ends were used to amplify each sample. The tails, which were used to facilitate addition of barcode and Illumina flow cell adapters in the second PCR (step 2), consisted of a tag sequence (priming site for the second PCR), a variable-length spacer (to increase sequence diveristy), and a 2bp linker (to separate the target specific primer from the rest of the tail). Each primer used for the second PCR consisted of the Illumina adapter sequence joined at its 3’ end to an 8bp Hamming error-correcting barcode; each amplicon library, consisting of DNA fragments from one soil sample, had a unique pair of bar codes.
