# Supplementary material for: Characterization of soils conducive and non-conducive to Prunus replant disease
Source: PLoS One. 2021 Dec 10;16(12):e0260394. doi: 10.1371/journal.pone.0260394 (PMC8664177; doi:10.1371/journal.pone.0260394)
Supplement: S2 Table — (DOCX) [file pone.0260394.s006.docx]

**S2 Table.** Diversity estimates of microbial community associated with PRD-inducing and non-inducing soils

| **Primer set** | **Soil type** | **Observed ASVs** | **Shannon** | **Pielou’s evenness** |
| --- | --- | --- | --- | --- |
| **Bacterial V3V4 region** | non-inducing | 493.22 ± 253.9 | 5.548 ± 0.755 | **0.914 ± 0.043** |
|  | PRD-inducing | 437.57± 175.8 | 5.646 ± 0.378 | **0.940 ± 0.011** |
| **Bacterial V5V7 region** | non-inducing | 126.519 ± 56.1 | 4.442 ± 0.515 | 0.934 ± 0.014 |
|  | PRD-inducing | 134.28 ± 71.05 | 4.446 ± 0.606 | 0.936 ± 0.021 |
| **Fungal ITS1 region** | non-inducing | 135.36 ± 43.95 | 3.546 ± 0.782 | 0.725 ± 0.128 |
|  | PRD-inducing | 128.19 ± 37.01 | 3.731 ± 0.499 | 0.774 ± 0.076 |
| **Fungal ITS2 region** | non-inducing | 95.52 ± 32.85 | 3.437 ± 0.611 | 0.761 ± 0.094 |
|  | PRD-inducing | 84.61 ± 26.67 | 3.355 ± 0.459 | 0.763 ± 0.066 |
| **Oomycete ITS1 region** | non-inducing | 29.59 ± 9.01 | 2.325 ± 0.36 | 0.713 ± 0.074 |
|  | PRD-inducing | 25.59 ± 8.668 | 3.23 ± 0.38 | 0.742 ± 0.065 |

Mean (±SD) of richness (Observed ASVs), Shannon diversity and Pielou’s evenness calculated for bacteria, fungi and oomycetes. The bold numbers represent the significant (*P*< 0.05) results of post hoc Kruskal Wallis test among non-inducing and PRD-inducing soil.
