# Supplementary material for: Characterization of soils conducive and non-conducive to Prunus replant disease
Source: PLoS One. 2021 Dec 10;16(12):e0260394. doi: 10.1371/journal.pone.0260394 (PMC8664177; doi:10.1371/journal.pone.0260394)
Supplement: S3 Table — (DOCX) [file pone.0260394.s007.docx]

**S3 Table.** NCBI Blast results for top ranked ASVs based on RF classification of bacterial, fungal and oomycetes features

| **Organism** | **ASV Labels****^a^** | **NCBI Blast** |
| --- | --- | --- |
|  |  | **Scientific name, accession number, and identity (%)** |
| **Bacterial ASVs** | V4_272 | *Gemmatimonas phototropica*, NR_136770.1 (88.14) |
|  | V4_86 | *Bacillus thuringiensis*, CP053938.1 (100) |
|  | V4_04 | *Pseudomonas sp.*, MT629836.1 (100) |
|  | V4_1194 | *Conexibacter stalactiti*, NR_157993.1 (95.65) |
|  | V4_297 | *Gaiella occulta*, NR_118138.1 (93.28) |
|  | V4_37 | *Gemmatimonas phototropica*, NR_136770.1 (89.45) |
|  | V4_1554 | *Acidicapsa ligni*, NR_116444.1 (81.64) |
|  | V4_426 | *Gaiella occulta*, NR_118138.2 (92.09) |
|  | V4_595 | *Azoarcus sp.*, CP022958.1 (96.84) |
|  | V4_269 | *Sphingomonas daechungensis*, NR_133862.1 (100) |
|  | V4_236 | *Devosia sp.*, JQ977621.1 (99.21) |
| **Fungal ASVs** | ITS1_227 | *Articulospora_proliferata*, MN898239.1 (98.86) |
|  | ITS1_65 | *Mortierella alpina*, MT514388.1 (100) |
| **Oomycetes ASV** | OOM1_13 | *Pythium sylvaticum*, MN541114.1 (100) |

^a^ Indicates the ASV labels assigned in the ASV tables of each microbial community.
