# Supplementary material for: Characterization of soils conducive and non-conducive to Prunus replant disease
Source: PLoS One. 2021 Dec 10;16(12):e0260394. doi: 10.1371/journal.pone.0260394 (PMC8664177; doi:10.1371/journal.pone.0260394)
Supplement: S4 Table — (DOCX) [file pone.0260394.s008.docx]

**S4 Table.** NCBI Blast results for top ranked ASVs based on RF regression of bacterial and fungal features

| **Organism** | **ASV Labels ^a^** | **NCBI Blast** |
| --- | --- | --- |
|  |  | **Scientific name, accession, and identity (%)** |
| **Bacterial ASVs** | V4_86 | *Bacillus thuringiensis*, CP053938.1 (100) |
|  | V4_26 | *Vicinamibacter silvestris*, NR_151905.1 (92.49) |
|  | V4_01 | *Bacillus cucumis*, NR_148626.1 1(00) |
|  | V4_95 | *Gemmatimonas phototrophica*, NR_136770.1 (100) |
|  | V4_66 | *Gemmatimonas phototrophica*, NR_136770.3 (88.54) |
|  | V4_457 | *Aciditerrimonas ferrireducens,* NR_112972.1 (88.76) |
| **Fungal ASVs** | ITS1_08 | *Dactylonectria macrodidyma*, MN988719.1 (100) |
|  | ITS1_28 | *Pseudoparmelia uleana*, KM657276.1 (74.19) |
|  | ITS1_43 | *Trichoderma virens*, MT530036.1 (100) |

^a^indicate the ASV labels assigned in the ASV tables of each microbial community
